# The Invisible Abuser: Attachment, Victimization, and Perpetrator Perception in Repeat Abuse

**DOI:** 10.1177/10778012251379423

**Published:** 2025-10-14

**Authors:** Mags Lesiak, Loraine Gelsthorpe

**Affiliations:** 1 Institute of Criminology, 2152 University of Cambridge, Cambridge, UK

**Keywords:** trauma bonding, Stockholm syndrome, battered women syndrome, attachment to perpetrators

## Abstract

Current models of trauma bonding often understate the perpetrator's role in shaping victims' emotional attachment, risking both victim—blaming and conceptual distortion. This study addresses that gap through interviews with 18 women who experienced repeat domestic abuse and sustained attachment to their perpetrators. The analysis identifies *attachment weaponisation* as a core mechanism of coercive control. Through grooming, trauma—sharing, and alternating cruelty with care, abusers deliberately manufacture emotional entrapment. These cycles don't merely mimic intimacy — they weaponise it, turning affection into a mechanism of control that binds victims to their perpetrators without the need for force or visible constraint. The findings also indicate a distinct perpetrator profile marked by volatility, manipulativeness, and performative vulnerability. The paper shows that trauma bonding isn't a symptom of weakness — it's a strategy of control. Abusers create attachment through manipulation, turning love into a tool of domination.

## Introduction

Understanding a victim's attachment to their abuser is not an easy task. This rather complex phenomenon does not suggest a common-sense explanation. Arguably, the lack of intuitive explanations makes it difficult to sympathize with the victim, giving rise to “why doesn't she leave him” attitudes, which Erin Pizzey (1974) explored 50 years ago in her book *Scream Quietly or the Neighbours Will Hear*. This way of thinking has personal, social, and political implications for how women with emotional attachment to their abusers are treated (Ahmad et al., 2018; Lahav, 2021). Those who remain with their perpetrators for love face not only regular mistreatment and exploitation at the hands of their abusive partners (Cantor & Price, 2007) but also exclusion, marginalization, and discrimination at the hands of public institutions and broader society (Pugh et al., 2021). The stigma attached to them means they are often left to face their problems alone, which makes them vulnerable and unsupported, fueling cycles of isolation and violence.

Some attempts to account for the romantic relationship between victims and their perpetrators include claims that women enjoy the abuse (masochism; Ghent, 1990), are too “helpless” to leave the abusive relationship (“learned helplessness”; Seligman, 1972), or “enable” perpetrators’ dysfunctional behavior for their gratification (codependency; Morgan, 1991). None of these theories was based on robust and reliable research that explored the experiences of women who report trauma bonding. Instead, these theories were based on Freud's controversial ideas (masochism), the behavior of electrocuted dogs (learned helplessness), and stories from Alcoholics Anonymous (AA; codependency).Moreover, none of those mentioned above examined the role of the perpetrator in the process of forming and maintaining the victim's attachment to their abuser. Instead, all these theories propose in one way or another that the victim is to “blame” for developing feelings for the abuser and associating the victim's romantic attachment to the perpetrator with an internal defect of personality by portraying her as “masochistic,” “helpless,” or “codependent.” If society approached pedestrian traffic accidents similarly, drivers would rarely be investigated or held accountable, with the assumption that pedestrians were always at fault. Such an unreasonable approach could only be justified if there was a widely accepted belief that pedestrians were inherently problematic or that drivers were too skilled to be considered potential causes of accidents.

Scholars have only recently started exploring perpetrators’ role in forming attachments with victims. Doychak and Raghavan (2020) introduced an innovative perspective by conceptualizing these attachments as “trauma-coerced,” aiming to mark a shift away from victim-blaming narratives. They offer a thorough operational definition of trauma bonding, examine how coercion contributes to it, and advocate for aligning with *DSM* criteria to provide legal avenues for victims who face criminalization (Doychak & Raghavan, 2023).

## Love in Captivity

An alternative position regarding attachment to the perpetrator was developed based on hostage research. This line of research has focused on situations that involve physical captivity (victim has limited or no power to leave), namely child abuse (De Young & Lowry, 2013); hostage situations (Cantor & Price, 2007); human trafficking (Egu, 2018); and cults (Ward, 2000). These ideas originated from the events that took place in 1973 during a bank robbery in Stockholm, Sweden. Four people were taken hostage, and after being released, the hostages defended their captors and would not agree to testify in court against them (De Fabrique et al., 2007). The victims’ peculiar behavior drew public attention, and soon after, the media started using the term “Stockholm syndrome” to describe victims’ emotional attachment to their perpetrators (Namnyak et al., 2008). Stockholm syndrome is also known as “trauma bonding,” especially when applied to settings other than hostage situations (Reid et al., 2013).

Casassa et al. (2022) reviewed definitions of trauma bonding currently available in academic literature. To mention a few: “the invisible strong emotional tie that develops between two individuals, where one person frequently harasses, beats, threatens, abuses or intimidates the other person” (Hopper, 2017; as cited in Sanchez et al., 2019, p. 49); “a paradoxical psychological phenomenon in which a positive bond between hostage and captor occurs” (Annitto, 2011; as cited in Lopez & Minassians, 2018, p. 264); and “a form of coercive control in which the perpetrator instills in the victim fear as well as gratitude for being allowed to live” (U.S. Department of Health and Human Services, n.d., as cited in Jordan et al., 2013, p. 361).

A widely cited early theoretical account of trauma bonding by Dutton and Painter (1981) posits that the victim's romantic attachment to the perpetrator arises from a recurring, cyclical pattern of abuse. Three main factors are involved in the establishment of a trauma bond: (a) a power imbalance, (b) intermittent reinforcement of good and bad treatment (reward and punishment), and (c) captivity. However, research shows that captivity and power imbalance are present in nearly all long-term abusive situations (domestic violence [DV], sexual exploitation, kidnapping) (Walklate, 2012). Still, only a few victims—an estimated 8% of more than 1,200 federal, state, and local hostage/barricade cases recorded in the FBI's Hostage/Barricade System—reported any features consistent with Stockholm Syndrome, and this figure comes from research conducted in the late 1990s (Fuselier, 1999). Since then, no subsequent research has been produced to establish or update these figures, leaving a gap in the current understanding of trauma bonding prevalence. Moreover, the theory cannot explain why some people who do not face captivity or a power imbalance also might report strong emotional attachment to their abusers.

To address some of these limitations, this study involved interviewing 18 women who were no longer involved with their abusive partners. All reported being financially independent during the relationship, often living separately from their abusive partners, and did not endure any form of physical captivity or safety threats upon ending the relationship. Most participants had been abandoned by their abusers and expressed a strong desire to reunite with them.

## Research Aim

To explore perceptions and experiences of women who face repeated abuse and attachment to their abusive partners, with a particular focus on how they perceive their perpetrators and the role these perpetrators play in forming and maintaining romantic attachment.

## Methodology

### Participants

In total, 18 participants were interviewed. All participants were female, aged 26 to 60. The following criteria were used to recruit the sample: (a) Female and living in the United Kingdom, (b) Financially independent, (c) Declaring repeat domestic victimization, romantic feelings for the abusive partner, and remaining in the relationship due to these feelings only and no other reasons.

To recruit an adequate number of participants, a research advertisement was placed on social media platforms, and Broxtowe Women's Project—a charity in Nottingham which supports victims of DV was approached and asked to circulate information among its users. In total, 34 females expressed interest via these two sources, and 18 females participated.

Only women who did not report any external constraints (financial, cultural, and social difficulties) or threats from the abusers were included in the sample. Before the interview, potential participants were asked the following screening questions: “Are there any other reasons that make you want to be with your partner apart from your romantic attachment to him? For example, difficulties that could relate to your financial, cultural, social situation, threats from your partner, or mental health and addiction issues?” If the participant answered yes, she would not be included in the sample. However, all potential participants declared that they did not experience any external pressures that could impact their feelings for their abusive partners, as the research advertisement specified inclusion criteria.

All participants were British women: two from Asian and Pakistani backgrounds, one from a Polish background, and the rest would describe themselves as white British. Participants’ occupations included: science teacher, dental nurse, dentist, well-being therapist, singer, manager in a charity, care assistant, general practitioner, chef, two nurses, copywriter, executive assistant, drug recovery worker, makeup artist, programmer, advertising manager, and a consulting analyst.

The study involved in-depth, semistructured Zoom interviews lasting 2–3 hr each, which were audio-recorded and transcribed using Otter. The author of this article conducted all interviews, and data were retrieved exclusively from these sessions.

### Materials: Interview Schedule

A semistructured interview schedule was developed for the study. It included 10 open-ended, nonleading questions on victims’ feelings and perceptions of their abusive partners. The order of the questions was chosen sensitively, starting with general questions like “How would you describe your partner?” and proceeding to more personal ones like “How does it feel to be attached to someone who hurts you regularly?” ending with emotionally neutral questions “How do you feel when you’re not around him?”

### Operationalization and Measurement of DV and Adverse Childhood Experiences

DV was explored through detailed narratives and experiences shared by participants during 2–3-hr semistructured Zoom interviews. It was operationalized as any form of physical, psychological, or emotional abuse inflicted by intimate partners. This encompassed behaviors like physical assaults, verbal threats, coercive control, and emotional manipulation. Measurement involved capturing and analyzing these narratives qualitatively, focusing on themes, patterns, and the subjective experiences of participants regarding DV.

Adverse childhood experiences (ACEs) were explored and understood through participants’ narratives during the interviews. The study focused on identifying and documenting various forms of adversity experienced by participants before age 18. Measurement involved qualitative analysis of these narratives to uncover specific instances of abuse (physical, emotional, sexual), neglect (physical, emotional), and household dysfunction (such as substance abuse, mental illness, and DV).

Rather than using standardized questionnaires, the study relied on participants’ self-reported accounts to qualitatively assess the prevalence and impact of ACEs within their life histories.

This approach allowed for a nuanced exploration of how ACEs were perceived and experienced by participants, providing rich qualitative data to understand their potential role in shaping later experiences, including instances of DV.

### Ethics

Ethical permission of the Ethics Committee of the Institute of Criminology at the University of Cambridge was obtained. Participants were alerted to the sensitive nature of interview topics before the interviews. Specifically, before we started each interview, they were given them the following trigger warning:

As you know, during the interview, we will be talking about your experiences in romantic relationships. You reported that you were in a long-term relationship with someone who often made you feel hurt. You may find some questions upsetting so please consider whether you can participate at this time.

The participants were informed about the interview's aims, risks, and benefits and their right to withdraw at any time. Participants were informed about available support, such as links to charities supporting DV victims, mental health services delivered by the NHS were provided, and help with a referral was offered if needed. Information sheets and the consent forms were emailed to the participants 2 weeks before the interview. Subsequently, participants were each asked to validate their interview transcript.

To protect participants’ privacy, the names used in this article are not their real names but pseudonyms chosen by the participants themselves. Participants were not asked about their real names. Therefore, no personal details were recorded, ensuring anonymity.

### Data Analysis

Each interview produced approximately 63 typed pages of transcribed data. The identification of a theme was a gradual process that followed Braun and Clarke’s (2006) six steps of analysis: (a) familiarization, (b) coding, (c) generating themes, (d) reviewing themes, (e) defining and naming themes, and (f) writing up. The transcripts were read multiple times, coding each line of the text and underlying it with different patterns of passages that appeared to share a theme. After familiarization with the data, the transcripts were processed using NVivo software analysis program; the NVivo codes were converted into process codes (see Appendix A for visualization of the coding framework, pp. 31–32). These were grouped, creating categories. Approximately three categories created a descriptive subtheme that finally converted into a theme that reflected the victim's perspective on the situation. Thematic saturation was achieved after the 12 data sets were analyzed. However, the rest of the interviews contained important information that enriched the picture of trauma bonding.

## Findings

Three themes were identified, which illustrate victims’ perceptions, feelings, and thoughts on their abusive partners and the role these perpetrators play in shaping the attachment:

1. “My two-faced ‘soulmate’”
2. “His and my childhood trauma”
3. “You're my drug: The magnetic pull I feel for you.”

### Theme One: My Two-Faced “Soulmate”

Participants were asked to describe their partners, and all painted a picture of someone full of contradictions: “charming,” “loving,” and “fun” on one hand but “abusive,” “narcissistic,” and “cruel” on the other. These accounts align with earlier research by Stark (2007) and Dutton and Goodman (2005), which suggests that such positive behaviors are often strategically employed to build deep emotional connection and dependency, making the victim feel uniquely understood and valued. In our participants’ experiences, this facade was repeatedly punctuated by episodes of abuse, narcissism, and cruelty, creating a cycle of unpredictability that intensified their emotional attachment—a pattern consistent with the mechanisms described in the earlier literature. The juxtaposition of warmth and cruelty reinforces the trauma bond, as victims become psychologically trapped in the hope of regaining the abuser's affection while simultaneously fearing their wrath. This duality, noted in previous research (Dutton & Goodman, 2005; Walker, 2009), contributes to confusion and entrenches emotional entrapment. Participant accounts in this study reflected this pattern, as Jane described:

(He was) outgoing, popular, funny, sometimes caring. How much detail do you want? Like he was also quite reckless, impulsive, and I suppose, unaware or uncaring how his actions affected anyone else.

When probed by the researcher about the fact that there were two sides to her partner, Jane responded:

Yeah, definitely. Definitely one super loving, really caring kind of nurturing as well. Really family orientated on one side that was kind of the perfect person to be in a relationship with, but then on the flip side of this, there was the impulsive sort of reckless person who you know, thought about what he wanted and just went after that. And didn't really take into account anybody else.

A similar statement was given by Lily, who talked about two dramatically different types of behavior that she experienced from her abusive partner:

Charming. Charming, charming. This is the first word I think about … Very charming. Very attractive. Always friendly. Always offering to buy a drink so seemingly generous. Always very complimentary. But then, as we got into a relationship, very secretive, very secretive. Always. Always quite distant about where he was and who he was with … I could talk to him about anything provided, and it wasn't about him … And actually don't feel that I know him any better than when I met him over 20 years ago.

To describe her abusive partner, Lily used the word “charming” 3 times in a row. This epizeuxis^1^ may indicate the intensity of her experience, emphasizing the importance of his charm in the relational dynamic. The Oxford English Dictionary (1989) defines the adjective “charming” as “very pleasant or attractive.” However, the second definition of a verb goes as follows: “use one's ability to please and attract in order to influence (someone),” capturing Lily's experiences more accurately. This dichotomy of character was also reported in Sophia's statement:

I sometimes kind of say he's a bit like Jekyll and Hyde. So now I've kind of learned about him. For the first year we were together, what a year and a half. It was everything that I wanted in a partner, as far as I can see. Didn't really see or notice any red flags. And then, toward the end, I felt like I was on eggshells … I was completely in love with him. He wanted all the same things as me … On the flip, he would also do abusive things.

These contrasting behaviors exhibited by the participants’ abusive partners left them feeling “confused,” “disoriented,” and “emotional”:

I think a whole lot of things that were happening—it's hard to describe him. I guess he was confusing me. (Sophia)

Olivia described how her partner kept her disoriented, and only later she realized it was not normal behavior:

Yeah, accepting the fact that all of this stuff wasn't normal, or almost was like a lightbulb moment, but it was always a bit like asking myself, ‘why did I ever put up with it all?’ I know; it was a weird one. It was always confusing. As I felt confused a lot of the time.

Some participants felt that their partners’ unpredictable and abusive behavior was their fault, implying an underlying feeling of guilt despite being the victim of the abuse:

I loved him so much; we were so happy and suddenly, he would just switch. I spent hours thinking if I did something to make him switch. When and where did I go wrong? (Luna)

Participants’ confusion and willingness to take the blame for the perpetrators’ abusive behavior might be related to the intense feelings of happiness experienced in the relationship. One participant stated that the happy moments with the perpetrator were so intense that her nonabusive earlier marriage of 12 years could not compare with that:

I can look back on 12 years of marriage, and there's nothing that stands out that makes me feel like a really happy moment of my life. I can look back on a toxic relationship and label 100 things that made me feel really happy. (Amelia)

A similar statement was given by another participant who said that after the abuse, the perpetrator would offer her comfort and “everything (she) ever wanted”:

Anna: He will be abusive controlling, and then he would be much of a comfort to you when you were anxious.

Researcher: How did you make sense out of this experience?

Anna: I don't think you do. I think that's the thing that is that cognitive dissonance of contradicting thoughts in your mind. I know in the beginning you go, you keep going back, because when you pull away, they're going to give you, give me everything I wanted.

A strong pattern of flattery alternated with abuse emerges. First, the perpetrator portrays himself as trustworthy and caring, probably to deceive the victim and establish an emotional bond that later serves as a tool of manipulation (Sinnamon, 2017). This behavior is in line with the literature on psychopathy and narcissistic grooming that is often discussed in the context of offending and personality disorders (Hare, 1970). In the second stage, abusive behavior occurs which then disorientates the victim, often resulting in self-blame that could be explained by the cognitive dissonance theory (Festinger, 1962). The victim may minimize the abuse or blame herself for preserving the image of the abuser created at the beginning. The present findings are aligned with previous research (Reid et al., 2013; Stark, 2007), suggesting a unique and impactful role of Intermittent Reward and Punishment (IRP) in forming and maintaining trauma bonding.

### Theme Two: His and My Childhood Trauma

All 18 participants disclosed experiencing ACEs when prompted to reflect on their childhood years, despite the interview questions being broadly phrased (e.g., “How was your childhood?”) and not specifically focused on childhood trauma. Moreover, all participants, when asked about their perpetrators’ childhood, stated that their abusive partners experienced childhood trauma as well.

The abuse the participants experienced as children included mental and sexual abuse, maltreatment, abandonment, rejection, and coercive control. One participant commented on experiencing a long-term illness, while the rest of the participants reported relational mistreatment:

When I was 16, I moved out of home because I couldn't stand the violence anymore in the house. I'd already tried to run away from home a few times. And my dad just got the police and asked them to take me into care. My grandma would always stick up for him. Always. I will say many times (to my grandmother): ‘I can't live like this, you know; it's him or me,’ and she would choose him every time. (Amelia)

Ava implied horrific abuse at the hands of her mother's partner: “My mother's boyfriend was doing everything he wanted with me, and I was only a child.”

Some participants perceived ACEs as the source of their tendency to trauma bonding:

Yeah, I've done a lot of thinking about it (the participant's childhood) and I think this is probably one of the reasons why I do experience these relationships. My mom raised me on her own, and she was very like, you know … she had depression when I was a child. So, she was very much like (long pause) unkind towards me when she was in those down days. So, I would stay a lot in my room, things like that. And then she would drink a lot. (Inga)

Participants’ descriptions of their partners’ early life experiences were similarly distressing:

He and his mom were extremely toxic—extremely toxic, like I saw him hit her. I saw her hit him in front of his younger siblings. There was a lot of jealousy of his older brother. So, his older brother lived with his dad. He lived with his mom and his younger siblings. And he was so jealous of his brother, his older brother having a relationship with his dad. And because he just didn't want a relationship with his dad, he was quite bitter that they split up and things like that; his dad moved on and got with another woman quite quickly and had his own family. There was a lot of bitterness around that. And like, it was, honestly, him and his mom was constant. (Anna)

Maya's abusive partner experienced particularly severe trauma: “He was brought up in an orphanage, abused in every possible way.”

Kim also talked about a significant incident that her abusive partner experienced as a child: “He watched his sister die, and he was only ten years old. He was holding her in his arms when she was choking.*”*

One of the participants described a scene in which her partner would argue in front of her kids that his childhood experiences were much worse than hers, although she lost her mother to murder:

My family, my children, were having a discussion, and they were just talking about, you know, my mom, you know, being killed. And they just went to mention my mom; he would always go, yeah, my childhood was harder than yours. You know. My childhood was always harder. It's like nobody could have had it worse than him. (Amelia)

Shared experiences of childhood trauma can foster emotional connection by reducing isolation and shame, often through processes of normalization and validation (Festinger, 1954; Platt & Freyd, 2012). However, the data suggest that abusers frequently exploit this emotional intimacy as a tool of control. As Amelia reflects, her partner routinely recentered his own suffering, invalidating her pain and manipulating others through guilt—a reversal of empathy that weaponizes shared trauma. Similarly, Inga describes how her partner disclosed her childhood experiences in front of friends, deepening her shame and contributing to social isolation: “With hindsight, I think he did it to distance me from them.”

Anna's account further illustrates this dynamic: “We shared the same pain … but it was just an illusion so he could keep me around.” These narratives reveal how perpetrators co-opt the healing potential of mutual trauma to justify abuse, foster dependency, and obscure responsibility.

### Theme Three: You’re My Drug: The Magnetic Pull I Feel for You

Toward the end of the interview, participants were asked about their feelings for the abuser and their perception of their overall relationship. Many of the participants compared their situation to addiction, in which they feel a compulsion to be with the perpetrator despite the mistreatment they experience. They expressed disparity between what they think and feel, cognitively understanding that the relationship is harmful to them, but their effect “pushes” them back to the abuser.

Participants often expressed a significant disparity between their cognitive understanding and their emotional responses. While they intellectually recognized the harmful nature of the perpetrator, their emotions—shaped by the abuser's intermittent reinforcement—compelled them to return. This emotional pull can be explained by the dynamics of trauma bonding, where the victim's attachment is strengthened not just by positive moments but by the resolution of tension following abusive episodes (Dutton & Painter, 1993). The tension and its resolution create a powerful psychological reward system akin to the highs and lows experienced in addiction, making the victim feel temporarily relieved or even euphoric when the abuser shifts back to being “charming” or “loving.”

One participant reported that her feelings are so strong that she would do anything to be around her abusive partner:

Even though I can write a list and I can, I can logically look at it … (but) it's literally like saying to a heroin addict, if you keep taking heroin, you're gonna die. And if I keep being in this situation, it just seems the trend is showing it's just gonna get worse. And I'm not going to get the things that I want in life: a family, a partner—but here I am. It really is what you know, like when people say they're on drugs and you know, they steal, they do anything. I can resonate. So, it feels like there isn't anything I wouldn't do to get it. Like, like drugs, pretty much. (Ave)

Three of the women interviewed stated that their compulsion to be around the perpetrator was so strong that they relocated to different cities to prevent themselves from having contact with him. As Amelia mentioned:

I needed to remove myself from that sort of temptation or those encounters or bumping into him (audio not clear) before I was at risk of seeking that out. It's like an addiction, you know, and since that choice has been taken away by moving all the way here. The last few weeks, I have felt free. But I did have to drive back to go see my eldest child, and I had to pull in at a petrol station. And even that being in the vicinity of a couple of miles from where we live caused me massive anxiety … I'd be scared if I'm being truthful and honest. I'd be in fear of having contact with him. And that would lead to more contact with him because I'd be frightened of slipping back into that again. So, the anxiety is if I see him, I mean fear of breaking my boundaries or letting myself down. So, it does feel like it did; it just feels like an addiction.

Another participant, Ali, mentioned leaving the capital and moving two and a half hours away to prevent her from seeing her abuser. However, this did not entirely help since: *“*That still hasn't changed the interaction in terms of me being able to leave for definite.”

One participant went to great lengths lying to her own family to be able to move away from her abuser:

As soon as I finished my master's, I moved away. My parents thought I got this amazing job offer, and that's why I left, but the truth is I was looking for a job far away from him. (Emma)

Emma also clarified that she had to move away because if she had stayed, she would have been “right back with him” as “it is stronger than her.”

One of the participants describes the process of breaking up as “torture”:

I couldn’t sleep for months after we broke up; I was constantly stressed, in pain, craving. It was honestly like coming off the drugs; it was hell, it was torture. Being without him was worse than the abuse. But *somehow,* I took the pain, and after a few months, the pain stopped, and I’m free now, but it hurt as hell. (Moon)

The participant used the word “craving” to describe how much she wanted to reunite with her abusive ex. “Craving” is often used within the context of drug withdrawal to capture the intensity of wanting. This choice of wording may indicate the compulsive nature of proximity seeking the participant experienced at that time.

In this context, the victim's cognitive recognition of the pain she experiences clashes with the deep-seated emotional responses inflicted through coercive control (Stark, 2007). Herman (2015) suggests the grip of trauma bonding is rooted in a profound disconnection between understanding and feeling. Victims may intellectually comprehend the dangers of their situation, yet the emotional reinforcement of hope—coupled with fear of abandonment—creates a paradox that complicates their ability to leave. Thus, the interplay of coercive control, the cycle of violence, and intermittent reinforcement forms an emotional trap that appears to resemble behavioral addiction in nature. Victims are left navigating their situation with confusion and pain, struggling with the duality of their experience—the powerful pull of their emotional bonds contrasted with the harm they experience.

## Discussion

Participants in this study described their abusers as *confusing, intense, and omnipotent*. This perception arose from the perpetrator's contradictory behaviors—alternating between affection, charm, and care, and cruelty, control, and abuse. The findings highlight the centrality of IRP in the creation and maintenance of trauma bonds. In the accounts given, IRP was frequently coupled with grooming and the manipulation of shared trauma, creating an emotionally entrapping dynamic that participants likened to behavioral addiction. These results underline the need to shift theoretical and practical attention toward the *active strategies of perpetrators*, rather than victim deficits, when explaining the persistence of abusive relationships.

### Entrapment

The narratives in this study demonstrate that intermittent reinforcement alone can produce a powerful psychological bond, even in the absence of physical captivity or financial dependence. Women in this sample—financially independent and often living separately from their partners—still described an intense compulsion to return to the abuser. This suggests that captivity, while potentially intensifying dependency, is not a necessary condition for trauma bonding.

As Stark (2007) and Herman (2015) suggest, this form of manipulation fosters obsessive thoughts, deepens emotional dependency, and amplifies self-doubt, making it increasingly difficult for victims to recognize and resist the abuse. Exposure to inconsistent and unpredictable patterns of rewards and punishments leads to confusion and compulsion in the recipient, as observed in both animals (Skinner, 2019) and humans (Bechara & Damasio, 2005; Ferster & Skinner, 1957; Volkow et al., 2012) studies. Behaviors reinforced intermittently are more resistant to extinction than those reinforced consistently, resulting in more enduring behavior patterns (Ferster & Skinner, 1957). In domestic abuse situations, perpetrators often reward obedience and compliance, thereby strengthening the victim's dependence and reinforcing their subservience while exerting control and manipulation (Stark, 2007). As a result, it might be particularly challenging for victims to alter their obedient behaviors, which might be highly resistant to change due to the nature of the schedule they were exposed to, as shown by Ferster & Skinner (1957). Thus, understanding the role of intermittent reinforcement is essential for developing effective interventions and support mechanisms for those affected by trauma bonding.

### Shared Childhood Trauma Impacts Victims’ Perception of the Perpetrator, Often Resulting in a False Sense of Connection

Every participant in this study reported ACEs and described their perpetrators as having similar histories. This perceived commonality often fostered an initial sense of deep connection. However, participants’ accounts revealed that abusers frequently weaponized these shared experiences—invalidating the victim's suffering, recentering their own pain, or using personal disclosures to humiliate and isolate.

The data indicate that this tactic operates within the IRP framework: shared trauma was invoked to establish closeness during the “reward” phase and later to justify or excuse cruelty in the “punishment” phase. This mirrors descriptions of grooming tactics that exploit victim vulnerabilities (Lanning, 2000; Stark, 2007) and perpetuate a cycle of psychological and emotional control (Salter et al., 2003). Sinnamon's (2017) Seven-Stage Model of Adult Grooming—encompassing victim selection, gaining trust, meeting needs, priming, instigating abuse, and exerting control—maps closely onto these accounts. In this study, participants described how “loving and attentive” partners in the early grooming stages gradually shifted into “cruel and exploitative” abusers, embedding shared trauma narratives within a broader coercive control strategy.

Experiencing childhood trauma also appeared to heighten vulnerability to such tactics. Unresolved trauma can erode self-esteem (Briere & Jordan, 2004), lower standards for acceptable treatment, and make it more difficult to recognize or challenge abusive behavior (Herman, 2015). This compromised boundary-setting renders individuals more susceptible to manipulation, particularly when abusers present shared suffering as evidence of intimacy and understanding.

### The Process of Subjugation

Research has demonstrated biological, emotional, and behavioral parallels between addiction and attachment, even in healthy relationships (Burkett & Young, 2012; Fisher et al., 2016; Insel, 2003). Compulsive proximity-seeking behaviors resembling addiction can occur in secure, nonabusive relationships (Millings & Walsh, 2009), suggesting that the neurobiological mechanisms underpinning attachment—particularly those involving opioid and dopamine pathways—are part of a broader human tendency toward social bonding. The present findings indicate that perpetrators in abusive relationships deliberately exploit this tendency, amplifying it through IRP and grooming strategies.

In the accounts collected here, abusers intensified dependency by coupling cycles of affection and cruelty with the exploitation of shared trauma to create a false sense of intimacy. These tactics deepened participants’ emotional entrapment, making it increasingly difficult to recognize or resist coercive control. Many participants explicitly described their experiences as “addictive” or “compulsive,” underscoring how this dynamic felt less like a choice and more like a form of psychological captivity. Unlike healthy bonds, this attachment was cultivated and weaponized by the perpetrator, operating as a covert but pervasive mechanism of subjugation that was largely invisible to outsiders.

## Implications for Theory

This study reframes trauma bonding as a perpetrator-driven process, shifting explanation away from victim pathology toward the deliberate strategies used by abusers. The findings show that physical captivity or financial dependence is not necessary conditions for trauma bonding; instead, bonds can be manufactured through a “two-faced soulmate” profile in which intense affection alternates with cruelty. This dynamic integrates IRP, grooming tactics, and the exploitation of shared trauma into a coherent coercive control strategy, deliberately cultivating emotional dependency. Survivor accounts align with behavioral addiction models, suggesting neurobiological as well as psychological parallels. The study bridges behavioral conditioning, attachment theory, grooming theory, and coercive control, offering an integrated framework that identifies specific perpetrator tactics as critical drivers of trauma bonding and challenges assumptions about its prevalence and causes.

## Implications for Policy and Practice

These findings call for a reorientation of domestic abuse policy and practice toward recognizing and disrupting the deliberate strategies perpetrators use to create and sustain trauma bonds. Intervention frameworks, including risk assessment and safety planning, should account for tactics such as IRP, grooming, and the exploitation of shared trauma, rather than focusing solely on victim vulnerabilities. Eligibility criteria for protective services remain structurally tethered to visible forms of restraint. Survivors entrapped through weaponised attachment—where emotional dependence is systematically produced through cycles of reinforcement and withdrawal—are routinely excluded from recognition and response, not because coercion is absent, but because their captivity leaves no physical or financial trace. Such patterns of relational domination, frequently enacted through what this paper terms *strategic attachment conditioning*, mirror behavioural addiction models: they operate not through force, but through intermittent access to care, strategic contradiction, and the erosion of autonomous response. These dynamics are not symptoms of pathology, but artefacts of sustained coercive strategy.

Training and assessment frameworks should centre behavioural indicators—such as two-faced perpetrator presentation, IRP (intermittent reinforcement and punishment) cycles, and relational destabilisation—as core evidence of coercive control through attachment weponisation. Models drawn from behavioural addiction science, including pattern interruption, and trigger deactivation, could offer tools for support without invoking blame or psychologised failure. Legal definitions of coercive control should also evolve to reflect that entrapment can be manufactured without confinement.

## Notes

1. Repetition of a word or phrase in immediate succession.

## Appendix A. Coding Framework.

**Table A1.** Visualization of How Themes Were Created: An Overview of Nvivo Codes, Process Code, Categories, Subthemes, and Themes.

Nvivo Code	Process Code	Categories	Subthemes	Theme
‘things I wanted to hear’ ‘made me feel loved ‘giving me everything I wanted’ ‘Mind games’ ‘kind to everyone but me’ ‘Very self-centred, demanding, needy’ ‘contacted my parents started demanding things’ ‘Would make me’	Attracting the victim Fabricating love Grooming the victim Emotional manipulation Performing around others Exhibiting narcissistic behaviours Harassing victims’ family Forcing submission	The abuser produces the illusion of love to create emotional dependency in the victim The abuser begins exploitation and shows his abusive side. From grooming to extraction.	Victims subjected to emotional manipulation become confused and traumatised	**My two-faced 'soulmate'**
‘constant nightmares’ ‘abused for years’ ‘Don't want to remember my childhood’ ‘his trauma was worse than mine’ ‘he was nothing to his mum’ ‘no one loved him’	Reliving the trauma Subjected to long-term abuse as a child Repressing traumatic memories Seeing perpetrators’ experiences as more significant than hers Empathising with the abuser Feeling pity for the abuser	Victim struggles with unresolved childhood trauma The victim’s past experiences are reflected back by the abuser, creating a sense of connection that becomes a point of control.	Prior experiences of abandonment and harm are mirrored and manipulated	**Mine and your childhood trauma**
‘It’s like heroin’ ‘‘craving him’ ‘I would do anything’ ‘I can’t stop’ ‘Can't function without him’ ‘Terrified of being alone’	Perceiving the perpetrator as addictive Perceiving perpetrators as tempting Escalating desperation Limited control over oneself The perpetrator’s absence is experienced as disabling Fear weaponised against the victim	Engineered attachment Affective captivity	**connection is weaponised** and autonomy is eroded through **affective control**	**You’re like a drug; the magnetic pull I feel for you**
